# Glycomic-Based Biomarkers for Ovarian Cancer: Advances and Challenges

**DOI:** 10.3390/diagnostics11040643

**Published:** 2021-04-01

**Authors:** Francis Mugeni Wanyama, Véronique Blanchard

**Affiliations:** 1 Institute of Laboratory Medicine, Clinical Chemistry and Pathobiochemistry, Charité—Universitätsmedizin Berlin, Corporate Member of Freie Universität Berlin, Humboldt—Universität zu Berlin and Berlin Institute of Health, 13353 Berlin, Germany; francis.wanyama@charite.de; 2 Department of Human Pathology, Clinical Chemistry Unit, University of Nairobi, Off Ngong Road, Nairobi 19676-00202, Kenya

**Keywords:** ovarian cancer, biomarker, clinical biomarker, glycan

## Abstract

Ovarian cancer remains one of the most common causes of death among gynecological malignancies afflicting women worldwide. Among the gynecological cancers, cervical and endometrial cancers confer the greatest burden to the developing and the developed world, respectively; however, the overall survival rates for patients with ovarian cancer are worse than the two aforementioned. The majority of patients with ovarian cancer are diagnosed at an advanced stage when cancer has metastasized to different body sites and the cure rates, including the five-year survival, are significantly diminished. The delay in diagnosis is due to the absence of or unspecific symptoms at the initial stages of cancer as well as a lack of effective screening and diagnostic biomarkers that can detect cancer at the early stages. This, therefore, provides an imperative to prospect for new biomarkers that will provide early diagnostic strategies allowing timely mitigative interventions. Glycosylation is a protein post-translational modification that is modified in cancer patients. In the current review, we document the state-of-the-art of blood-based glycomic biomarkers for early diagnosis of ovarian cancer and the technologies currently used in this endeavor.

## 1. Introduction

In 2018, the Global Cancer Observatory GLOBOCAN reported an estimate of 295,414 ovarian cancer (OvCa) cases out of the total incidences of female cancers of about 8.8 million globally. Of the total incidences of death from female cancers of 4.1 million globally, 184,799 mortalities were from ovarian cancer. Further predictions indicate a worldwide increase of OvCa incidences to 404,268 and 268,302 deaths by the year 2035 [1]. OvCa exhibits a high amount of heterogeneity by the very nature of their histological or molecular origin. OvCas are classified into five major types based on their histological and molecular genetics. They are high-grade serous type, which is the most abundant, followed by endometrioid, clear cell, mucinous and the least abundant, the low-grade serous ovarian carcinoma [2,3].

The importance of diagnosing OvCa while still in its early stages is underscored by the benefits accrued to the patient that include increased chances of successful cure or prolonged survival period. A diagnosis made in the early stages allows sufficient duration of time to institute various mitigative therapeutic approaches with a 90% chance of cure [4]. Past research findings have shown that about 90% of patients diagnosed with stage I OvCa have a 5-year survival rate. Unfortunately, only about 30% of the women are diagnosed with stage I OvCa, whereas the majority are diagnosed in stages III and IV, with reduced 5-year survival rates of 20% and 6%, respectively. Important to note is that the patient’s survival rate has a strong correlation with the stage of the disease at which the diagnosis is made [5,6].

Diagnosis of OvCa in its early stage is impeded by the very nature of its unspecific symptoms whereby in the majority of cases, signs and symptoms become only clear when the tumor is in its advanced stages (FIGO Stage III and IV) [4,5]. Moreover, the lack of effective screening and early diagnostic strategies that can detect OvCa in its formative stages, before manifestation of full-blown symptoms, has been the missing link in the fight to reduce the mortalities associated with late-stage diagnosis [7]. Introduction of new, effective and less-invasive biomarkers that will help to detect OvCa promptly are, therefore, urgently needed.

Glycosylation is an essential protein’s post-translational modification that is deferentially reflected in an individual’s health or pathological status. Glycans are made up of monosaccharides that are linked by glycosidic bonds in many different ways to form highly branched structures, hence they exhibit enormous heterogeneity. This is in contrast to the highly conserved template-driven DNA and proteins [8]. Aberrant modifications of glycans have been reported for many forms of cancer and they correlate with the overexpression of enzymes that are responsible for the biological processing of glycans, namely glycosidases and glycosyltransferases [9]. They are usually protein-specific, cell-specific, site-specific and their mechanisms have been studied in detail; for a review, see [10]. In human blood, all proteins, except albumin, are glycosylated. *N*-Glycosylation occurs at the side chain of asparagine residues in nascent glycoproteins according to the consensus motif asparagine-X-serine/threonine, where X can be any amino acid except proline, while O-glycans are linked to the hydroxyl group of serine or threonine.

In the following, we review the clinically approved glycan-based biomarkers for OvCa, the milestones achieved in the ongoing glycomics research, as well as the state of technology used in the field and the challenges encountered.

## 2. Clinically Approved Blood-Based Glycan Biomarkers for Ovarian Cancer

Cancer antigen-125 (CA125) is a glycoprotein blood-based biomarker used for the detection of OvCa. It was clinically approved in 1981 and it is the major OvCa biomarker used routinely in clinical settings. However, CA125 is unreliable for the detection of early stage OvCa due to its associated limitations of low sensitivity and specificity. Other glycoprotein-based tests that have since received clinical approvals include human epididymis 4 (HE4), carbohydrate antigen 19-9 (CA19-9), and multi-parametric assays such as the risk of ovarian malignancy algorithm (ROMA), OVA1 and OVA2 [4,11,12] (Table 1). Multi-parametric tests find their clinical utility mostly in the risk stratification of the subjects into low- or high-risk groups.

### 2.1. CA125 (MUC16)

The CA125 mucin is a highly *N*- and *O*-glycosylated transmembrane glycoprotein expressed in the epithelium of Müllerian and coelomic types, namely ciliated cells, endometrium, endocervix and pericardium, peritoneum, mesothelial cells of the pleura, respectively [6]. The current CA125 automated ELISA test uses a combination of two monoclonal antibodies OC125 and M11 generated against non-overlapping domains of CA125 [13]. The concentration of more than 35 U/mL are suggestive of a possible ovarian malignancy [14,15]. It is the most widely studied blood-based biomarker for ovarian cancer and has been the gold standard routine biomarker for ovarian cancer ever since its approval by the FDA in 1981 [16] (Table 1). However, CA125 is neither specific nor sensitive as a diagnostic marker for ovarian cancer because it is found to be elevated in gynecological benign diseases affecting pre-menopausal women as well as in conditions such as liver cirrhosis and heart failure, thereby limiting its applicability as a biomarker for OvCa [4,15,17,18]. Furthermore, fluctuations in CA125 levels are also evident in physiological conditions such as pregnancy and menstruation hence, negating its importance as a diagnostic marker for OvCa [18,19]. Of importance to note is that about 20% of the epithelial OvCa cases of serous origin do not show overexpression of CA125 hence, the likelihood to miss out such cases when CA125 is used as the only biomarker [20,21]. The meta-analysis study of Ferraro et al., revealed an overall sensitivity of 79% and specificity of 78% [16]. Mixed results have been reported by subsequent studies [22,23]. CA125 is, therefore, neither a definitive test that meets expectations of an accurate early diagnostic marker for ovarian cancer nor a reliable screening tool for the general population. However, it is highly recommended for monitoring the effectiveness of the treatment responses and detection of relapse in the remission period by conducting serial measurements [4].

**Table 1:** Clinically approved glycosylated ovarian cancer biomarkers.

Marker	Detection Type	Sensitivity (%)	Specificity (%)	Clinical Use	FDA Approval (Year)	Reference
CA 125	Protein concentration	79–94	59–82	Monitoring therapy and relapse	1981	[16,22,23]
HE4	Protein concentration	64–71	85–96	Monitoring therapy and relapse	2008	[12,22,24]
CA 1-9	Protein concentration	50–53	84–97	Monitoring therapy and relapse	2002	[25,26]
ROMA test	Protein concentration	91–94	75–84	Prediction	2011	[18,22,27]
OVA1 test	Protein concentration	77–96	28–35	Prediction	2009	[28,29]
OVA2 (Overa)	Protein concentration	91	69	Prediction	2016	[30]

### 2.2. Human Epididymis 4 (HE4)

Human epididymis protein 4 (HE4), a secretory product of the WFDC2 gene, is a glycoprotein that originates from the epithelial cells of the human epididymis, which is overexpressed in ovarian tumors [18,28]. It is an 11 kDa small secretory glycoprotein with hydrophobic amino acids at the terminus, expressed in patients with endometrioid, clear cell, epithelial, and mucinous ovarian cancers [31]. HE4 was approved by the FDA in 2008 for use in monitoring patients with an established diagnosis of ovarian cancer but not for screening early stage OvCa in asymptomatic women [6]. Unlike CA 125, HE4 values are upregulated in mucinous ovarian malignancy, giving it a wide detection scope for the subtypes of OvCa. Furthermore, its values are not influenced by common benign gynecological and medical conditions, as is the case with CA 125, hence they are better in terms of specificity [31]. Moreover, previous research findings demonstrated that the HE4 level is elevated in over 50% of ovarian tumor cases, which otherwise show normal expression of CA 125. Hence, HE4 exhibits superior sensitivity and specificity for detecting early stage OvCa as well as discriminating OvCa from benign ovarian tumors compared to CA 125. HE4 also performs better in premenopausal women as well, compared to CA125 [20,28,32].

When CA125 and HE4 tests were used as a dual marker, improved diagnostic performance characteristics were registered. Moore et al. reported the analysis of the combined premenopausal and postmenopausal patients with benign neoplasms, or with cancer or low malignant potential (LMP) tumors using the dual marker algorithm, and found a sensitivity of 86% at a specificity of 74.7%. While using the dual marker in postmenopausal women, a sensitivity of 92.5% at a specificity of 74.7% was reported and finally, in premenopausal women only, a sensitivity of 67.4% and specificity of 74.8% was obtained [18]. When distinguishing benign ovarian tumor from early stage ovarian cancer, Nolen et al. reported improved sensitivity and specificity of 74.2% and 85%, respectively, when CA125 and HE4 tests were used as a dual test [33]. Moore et al. reported a sensitivity of 72.9% at a specificity of 95% for the HE4 test when used alone to discriminate benign ovarian tumors from OvCa. While at the same specificity of 95%, CA125 produced a sensitivity of 43.3%. Improved sensitivity of 76.4% at a specificity of 95% was reported when the two tests were done as a combination [12].

### 2.3. CA 19-9

Carbohydrate antigen 19-9 (CA19-9) is a tetrasaccharide carbohydrate also named sialyl Lewis^a^ (*N*-acetylneuraminic acid-α2-3-galactose-β1-3[fucoseα1–4]-*N*-acetylglucosamine) that belongs to the larger family of mucinous markers secreted by human pancreatic and biliary ductal cells. Other sources of CA19-9 secretion are colon, gastric, endometrial and salivary epithelia [34]. CA19-9 is an established marker for pancreatic ductal adenocarcinoma, which received FDA approval in 2002. It is also found to be elevated in ovarian tumors, particularly the mucinous type [26,34]. However, its clinical utility is hampered by its insufficient sensitivity and specificity to discriminate early cancer from benign diseases [34,35]. One of the reasons for this is that α1-4-fucosyltransferase is not produced by about 6% of Caucasians and 22% of non-Caucasians [34]. A number of studies have suggested the use of serum CA-19-9 as a diagnostic marker for benign dermoid cysts, where they were successfully correlated with the tumor size, bilateral tumor involvement, and tumor torsion with CA19-9 values [36,37]. To this end, various studies have shown mixed results of CA19-9 analytical performance as a marker of OvCa. Santotoribio et al. reported a sensitivity of 50% with a specificity of 97% for the CA125. When they used CA19-9 and CA125 as a dual marker, an improved sensitivity of 66.7% at a specificity of 95% were obtained [25]. In another study, CA19-9 was able to detect mucinous ovarian cancer with a sensitivity and specificity of 52.7% and 83.8%, respectively [26]. Although previous studies have shown the usefulness of CA19-9 in predicting ovarian neoplasm of mucinous type, it was unable to differentiate benign, from borderline or malignant tumors [38].

### 2.4. Risk of Ovarian Malignancy Algorithm (ROMA)

ROMA is a multi-variable screening test designed by Moore et al. and approved by the FDA in 2011 for predicting the risk of ovarian malignancy in women presenting with pelvic masses [27]. It is a combination of CA125, HE4 and the menopausal status of the subject. Using this score, patients presenting with pelvic masses are categorized either as high or low risk to malignancy [27]. ROMA produced impressive performance characteristics for detecting ovarian cancer in a combined analysis of the pre-and postmenopausal women with a sensitivity of 93.8% and specificity of 74.9% [27]. In another study that involved patients with invasive and borderline ovarian cancer, ROMA produced overall sensitivity and specificity of 93.8% and 74.9%, respectively. On the other hand, premenopausal patients had a sensitivity of 100% and a specificity of 74.2% for detecting OvCa, while the postmenopausal had a sensitivity of 92.3% and a specificity of 76% [27]. Subsequent studies have provided mixed results [22,39,40].

### 2.5. OVA1

The OVA1 screening strategy is also a multi-variable index biomarker approved by the FDA in 2009 to distinguish malignancy from pelvic masses. It is a combination of five serum protein assays namely, second-generation CA 125-II, transthyretin, transferrin, β-2-microglobulin, and apoliprotein A1. The OVA1 test is useful in the stratification of the patients as low- or high-risk groups in terms of developing ovarian cancer [11,28,41]. OVA1 achieves its optimal potential when used as an add-on to physical examination and imaging. Improved sensitivity of 96% and a specificity of 35% were obtained when OVA1 was used along with the physical assessment of the patient by the physician [28]. Subsequent studies produced mixed performance characteristics [29].

### 2.6. OVA2

OVA2, also called Overa, is the second-generation test for OVA1. OVA2 is a multi-parametric test that stems from a combination of CA 125-II, HE4, apoliprotein A1, transferrin, and follicle-stimulating hormone. Coleman et al. reported improved performance characteristics from that of OVA1 with a sensitivity and specificity of 91% and 69%, respectively [30]. As a result, the FDA-approved Overa test in 2016 for screening women with pelvic masses, for either high or low risk of developing ovarian cancer [11].

## 3. Glycomics Methodologies

Glycomic studies can be approached at three different levels; by analysis of the free glycans, glycopeptides, or intact glycoprotein. The choice of the approach or strategy to use is dependent on various factors such as the nature and purity of the sample, level of technology vis-à-vis expert knowledge, and most importantly, the nature of information being sought. In general, glycan analysis consists of the isolation of free glycans or glycopeptides.

### 3.1. N-Glycan Profiling

Analysis of the released glycans is preferred when seeking to understand the compositional and structural characteristics of the total *N*-glycome. This is because of their conferred lower degree of complexity compared to glycopeptide or intact glycoprotein samples. Glycoproteins are deglycosylated either enzymatically or chemically. The tertiary structure of glycoproteins is first disrupted using proteases and/or denaturing reagents in order to make the glycosylation sites accessible. Enzymatic deglycosylation is achieved by treating a glycoprotein with endoglycosidases, preferably peptide-*N*-glycosidase F (PNGase F), which hydrolyzes the amide bond of the asparagine side chain for all types of *N*-glycans, except for those being α1-3 core-fucosylated. Endo H is used to specifically remove the high-mannose and hybrid structures [42]. On the other hand, Endo S is commonly used in immunological research questions as it specifically releases *N*-glycans of immunoglobulins (Ig) at asparagine-297 between the two *N*-acetylglucosamine (GlcNAc) of their reducing end [43]. Moreover, *N*- and *O*-glycans could also be released chemically, either separately or as a mixture of both, by hydrazinolysis through cleavage of the amide bonds under controlled conditions [44,45]. Alternatively, β-elimination is another chemical approach used to release both *N*- and *O*-glycans from the glycoprotein, but this technique is mostly employed on de-*N*-glycosylated proteins to release the remaining *O*-glycans. Sialic acids that terminate glycans can then be stabilized using permethylation, peracetylation, or dimethylamidation [45,46,47,48], which also improves ionization efficiency through increased sample volatility. Alternatively, the free reducing end of *N*-glycans may be labeled with a fluorescent tag, thereby increasing the sensitivity as well as the limit of quantitation and detection [45].

### 3.2. Glycopeptide Profiling

Glycopeptide analysis is the only bottom-up strategy that allows studying site-specific glycosylation. In other words, simultaneous analysis of glycan structures and the peptides they are bound on [49,50]. Prior to that, glycoproteins of interest are enriched using specific proteins or antibodies [51,52,53]. IgG, the most abundant immunoglobulin in human circulation is one of the most reported glycan-based biomarkers. Individual IgG subclass isolation consists of consecutive affinity purification using Protein A and/or Protein G. Protein A captures IgG1, IgG2 and IgG4, while Protein G, which is capable of capturing all IgG subclasses, can be applied at last to capture IgG3, after IgG1, IgG2 and IgG4 have been bound by protein A [54]. The resultant IgG preparation is then proteolytically digested by trypsin into glycopeptides, followed by purification using hydrophilic interaction liquid chromatography. Glycopeptide samples are subsequently profiled by mass spectrometry either in their native form or with derivatized sialic acids [52,54,55].

### 3.3. Profiling of Intact Glycoproteins

This middle-up approach is broadly used in research laboratories, clinical chemistry laboratories for routine diagnostics, or by pharmaceutical companies for product quality controls. Target proteins are analyzed either directly without purification or isolated by immuno-purification using specific antibodies prior to analysis [52,56,57,58]. Thereafter, glycoforms are analyzed by capillary electrophoresis, and also capillary electrophoresis coupled to mass spectrometry and hydrophilic interaction liquid chromatography coupled to mass spectrometry [57,59,60]. Clinical chemistry laboratories use the glycoforms of transferrin as a marker for alcohol abuse in adults and to detect congenital disorders of glycosylation in newborns [60]. The quality of biopharmaceuticals such as therapeutic antibodies, interferon-beta-1a, and human erythropoietin are verified after purification of the recombinant products [57,59]. Alternatively, intact glycoproteins can be purified with lectins, which are glycan-binding proteins. Glycoproteins resulting from the lectin pulldowns can be digested with proteases prior to analysis of the resulting glycopeptides [61].

### 3.4. Advances in High-Throughput Sample Preparation

The standard sample deglycosylation protocol using PNGase F enzymatic digestion takes about a day due to a time-consuming *N*-glycan cleavage step. The major improvement of high-throughput workflows lies in the fact that analytical turnaround times are reduced significantly. High-throughput workflows do not only reduce the time of analysis, they also significantly improved robustness and repeatability by introducing automation. They also support large-scale glycan analyses in 96- or 384-well-plates, which fosters compatibility between glycomics and clinical diagnosis [62,63].

Kronewitter and co-workers developed a high-throughput workflow for serum and plasma, where a microwave reactor was applied to hasten PNGase F digestion. Purification was subsequently performed using automated graphitic carbon solid-phase extraction and later subjected to mass spectrometric measurements of the *N*-glycans. Microwave-assisted *N*-glycan release takes about 10 min to achieve comparable levels of *N*-glycan release to the standard 16 h protocol [64]. Szabo et al. used the pressure-cycling technology to achieve the rapid release of glycoprotein *N*-glycans by PNGase F digestion. Pressure cycling technology alters protein conformation by forcing water molecules into the interior of proteins, causing them to unfold. On the other hand, cyclization between high and atmospheric pressure was shown to enhance the probability of endoglycosidase accessing the digestion site as a result glycan release is hastened [65]. In another approach, a reactor with immobilized PNGase F on monolithic polymer support in a capillary was used to allow the rapid and efficient release of *N*-glycans from IgG. Complete deglycosylation was achieved in 5.5 min at room temperature. The optimized reactor could also be integrated into a multidirectional system that is comprised of online glycan release, chromatographic separation and the mass spectrometric measurement [64,66]. Similarly, high-throughput 96-well plate protocols were developed for the isolation and analysis of IgG [54,67] and more recently, for IgA [54,67,68].

### 3.5. Analytical Instrumentation

There has been massive technological advancement in the last decade that led to the development of high-throughput methods. The most commonly used technologies include capillary electrophoresis (CE), ultra-performance liquid chromatography (UPLC) and mass spectrometry (MS).

Capillary electrophoresis is a relatively recent analytical platform for profiling labeled glycans. Glycan migration is mediated by an electric field, leading to the separation of positional glycan isomers and quantification can be achieved using labeled maltose [69,70]. The advantage of this technique is that it has high separation power, only requiring a small sample amount and with a quick turnaround time [71]. Glycan separation can also be performed on UPLC via hydrophilic interaction and fluorescence detection. Just like in CE, it is possible to resolve glycan isomers by UPLC. Retention times are standardized using an external glucose ladder [63] and a repertoire of elution positions for glycans has been made publicly available [72].

MS instruments are broadly used as well for glycomic studies. An MS instrument is made up of an ion source, which energizes the analyte, a mass analyzer that sorts the ions based on their *m/z* ratios, and a detector, which quantifies the resultant ions into a mass spectrum that is a plot of the relative abundance of the ions *m/z* ratio. Matrix-assisted laser desorption/ionization time-of-flight (MALDI-TOF) is a stand-alone instrument majorly used in the quantification of glycans or glycopeptides released chemically or enzymatically from the parent glycoprotein. It is the most frequently used soft ionization MS platform in glycobiology not only because of its good sensitivity but also due to its comprehensive analytical output. Several established research groups have extensively used this method in the sub-discipline of OvCa glycan biomarker discovery [73,74,75,76,77,78]. Unlike MALDI-TOF, UPLC or nano LC coupled electrospray ionization are used with mass analyzers such as quadrupole time of flight, orbitrap and Fourier transform ion cyclotron resonance mass analyzers.

The choice of one analyzer over the other depends on the analytical problem at hand. The factors that are considered in choosing the appropriate analyzer to use are the mass range, laser power, resolution, sensitivity, and ion transmission of the analyzer [79,80].

## 4. *N*-Glycosylation Traits Modulated in Human Serum of Ovca Patients

### 4.1. Total N-Glycome

**Figure 1:**
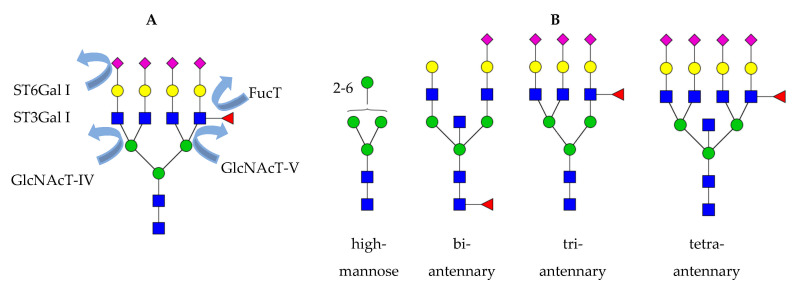
(**A**) Overview of the glycosyltransferases that are upregulated in cancer. GnT-IV, *N*-acetylglucosaminyltransferase IV; GnT-V, *N*-acetylglucosaminyltransferase IV; ST3Gal I, β-galactoside alpha-2,3-sialyltransferase 1; ST6Gal I, β-galactoside alpha-2,6-sialyltransferase 1; FucT, α1-3-fucosyltransferase. (**B**) *N*-Glycan structures that are of relevance in glycan biomarker studies on OvCa. At the level of total serum, high-mannose and biantennary bisecting *N*-glycans are downregulated, whereas sialylated fucosylated tri- and tetraantennary *N*-glycans are upregulated when primary OvCa patients as compared with controls. Besides, elevated bisecting *N*-glycans and some sialylated fucosylated tetraantennary structures have also been proposed as a marker for resistance to chemotherapy. Green circle, mannose; yellow circle, galactose; blue square, *N*-acetylglucosamine; red triangle, fucose; purple diamond, *N*-acetylneuraminic acid.

Aberrant modifications of glycans have been reported for many forms of cancer and always correlate with overexpression of enzymes that are responsible for their biological processing, namely glycosidases and glycosyltransferases, but also the sugar nucleotide donors and transporters [9]. Glycome modulations are usually protein-specific, cell-specific, site-specific and their mechanisms have been studied in detail; for a review, see [10]. Glycan modifications occur within tumors and their microenvironment (Figure 1). Abnormal glycosylation also occurs in the liver after receiving signaling from tumors [9]. As a result, modified glycosylation at the level of total *N*-glycome and acute-phase proteins measured in the blood (serum or plasma) is a prominent feature of ovarian cancer (Table 2). Inflammatory processes being also part of tumor development, variations of IgG glycosylation in the blood have also been noted.

High-mannose *N*-glycans are downregulated in OvCa, the same was observed for gastric cancer but not all types of cancer [74,78,81,82,83,87]. A decrease in hybrid structures was reported as well [78,82]; however, neither the molecular cause nor the role of high-mannose and hybrid *N*-glycans have been investigated in OvCa so far. Increased branching has been associated with the early stages of OvCa invasion and metastasis [73,74,76]. Branching is increased in OvCa due to the upregulation of mannoside acetylglucosaminyltransferases 4 and 5, genes that encode *N*-acetylglucosaminyltransferase IV (GnT-IV) and V (GnT-V). They catalyze the addition of β1-4-GlcNAc and β1-6-GlcNAc on the *N*- glycan core [10,84]. Branching leads to the formation of many tri- and tetraantennary structures that are fully galactosylated, which creates more substrates for the addition of terminal sialic acids [78]. An increase in tri- and tetraantennary structures is also associated with cancer progression due to its negative correlation with bisecting structures, which, in turn, are downregulated upon OvCa progression as observed in recurrent patients [76]. Indeed, the presence of tri- and tetraantennary structures do inhibit the action of *N*-acetylglucosaminyltransferase III (GnT III) that participate in the formation of bisecting *N*-glycans [88]. Interestingly, bisecting GlcNAcylated biantennary structures were associated with resistance to primary chemotherapy [85]. Increased branching is the reason for increased sialylation in OvCa and was associated with tumor progression and metastasis [76]. In human blood, increases in the sialic acid linkage ratio α2,3-/α2,6 were measured even in early stage OvCa [78] and correlate with increased ST3Gal I and ST6Gal I sialyltransferases measured in ovarian cancer tissues [89]. Increased fucosylation was observed in the form of sialyl Lewis^X^ (*N*-acetylneuraminic acid-α2-3-galactose-β1-4[fucoseα1-3] *N*-acetylglucosamine, sLe^X^) epitopes [73,74,76,81,82] and correlates with the expression of the corresponding α1-3-fucosyltransferase [90].

### 4.2. Immunoglobulin Glycosylation

Features of Immunoglobin G Fc glycosylation, especially galactosylation, reflect the body’s physiological (age, gender, pregnancy) or pathological state [91,92]. Galactosylation of complex-type *N*-glycans decreases with an increase in age, especially in women after menopause [92]. Glycome modulations also occur in autoimmune diseases, infectious diseases and malignancies including ovarian cancer [92,93]. These observed IgG glycan changes are most likely due to the inflammation occurring in the tumor and surrounding tissues as similar patterns are observed in rheumatoid arthritis [72]. In OvCa patients, a decrease in the levels of IgG galactosylation is observed [52,73,75] (Table 3).

Ruhaak and coworkers found differentially expressed IgA, IgG, and IgM glycopeptides in OvCa, whereby the IgG_1_ glycopeptide bearing a bisected biantennary *N*-glycan at asparagine-180 could efficiently discriminate EOC patients from healthy controls better than other Ig types [94]. In a study on subclass-specific IgG glycosylation in OvCa, it was found that IgG_1_ glycopeptide presented the greatest increase in agalactosylation and was strongly associated with CA125. Of interest to note in this study is that IgG_2_ and IgG_3_ were analyzed separately. IgG_3_ was more sialylated and galactosylated than IgG_2_ and consequently, was decreased in EOC patients [52].

### 4.3. Acute-Phase Proteins Glycosylation

Inflammation is a complex biological response to a stimulus such as infection, physical injury, cell damage or malignancy. During an inflammatory response, cytokines released from the site of inflammation travel to the hepatocytes to trigger an acute phase response that causes modification in the secretion and glycosylation of acute-phase proteins (APPs) (Table 4). The resultant concentration of APPs may increase by about 25% (positive acute-phase proteins) or may reduce by 25% (negative acute-phase proteins) [95,96].

The most abundant glycosylated APPs include haptoglobin, α-1 acid glycoprotein, α-1 antitrypsin, anti-chymotrypsin, fibrinogen, complement, fetuin, and transferrin. The changes in their *N*-glycan chains were studied for ovarian cancer by performing in-gel digestion of the corresponding 2D gel bands [73,97]. An increase in branching, core-fucosylation, sialylation, antennary fucosylation (named Lewis^X^ antigen, Le^X^) and antennary fucosylation on a sialylated branch (named sialyl Le^X^ antigen) were reported for haptoglobin, α1-antichymotrypsin, α1-antitrypsin, α1-acid glycoprotein, C1 esterase inhibitor and hemopexin [73,97,98]. Interestingly, the *N*-glycomes of transferrin, C1 esterase inhibitor, hemopexin and α2-HS-glycoprotein, which contain over 75% of biantennary structures, were not significantly changed in OvCa [73,97].

### 4.4. Diagnostic Performances of Glycan-Based Biomarkers

Several research groups have evaluated the diagnostic performances of glycan biomarkers with cohort sizes ranging from 58 to 299 individuals (Table 5). The GLYCOV score, integrating seven upregulated *N*-glycans and four downregulated *N*-glycans, demonstrated better diagnostic performance characteristics than the routine test CA125. At sensitivity of 97%, GLYCOV had a specificity of 98.4% for OvCa compared to the routine test CA 125, which had a specificity of 88.9% [74]. In another study addressing benign ovarian diseases versus early stage OvCa, GLYCOV had a sensitivity of 95%, whereas CA125 showed only 60% sensitivity [81].

A combination of a ratio of α-2,3-linked/α-2,6-linked sialylated structures and CA 125 to stratify early- and late-stage OvCa yielded improved sensitivity and specificity of 89.6% and 100%, respectively, compared to a sensitivity of 84.4% and specificity of 97% when CA 125 was used alone [78]. The candidate glycan-based biomarker developed by Leiserowitz and coworkers had better performances than CA125 with sensitivity and specificity ranging from 80–90% and 70–83%, respectively, whereas CA 125 had a sensitivity of 74% [77,83]. In another study of their working group, the IgG_1_ glycopeptide at asparagine 180 used in combination with CA125 improved the specificity from 86% to 95% in a validation cohort. When IgG_1_, IgA and IgM glycopeptides were combined with CA125, the sensitivity and specificity reached 96.2% and 92.3%, respectively [94]. The Gu laboratory showed that when IgG agalactosylation was used in addition to CA 125, the specificity could be improved by 19.4%, while the sensitivity was maintained at 90% [75].

### 4.5. Glycan Biomarker Traits for Ovarian Cancer Staging and Monitoring

Previous findings have shown the association between various modulated glycosylated structures with tumor growth and metastasis, which are key factors that inform cancer staging. Follow-up studies have also shown either reduction or increase of modulated glycan traits towards the normal baseline levels upon treatment [76]. The glycan traits that could potentially be helpful in OvCa staging and disease monitoring include sLe^x^, agalactosylation, galactosylation, branching (tri- and tetraantennary structures) and bisecting structures. Several research groups have shown how these modulated features of glycosylation in OvCa promote the invasiveness and metastasis of the disease [76,99,100].

It was previsouly shown that downregulation of high-mannose structures and upregulation of sialylated tri- and tetra-antennary structures containing mono- or difucosylation differentially expressed in stage I and stage II of the epithelial OvCa patients. GLYCOV value was able to discriminate OvCa stage I from stage II satisfactorily, whereas CA125 was not able to [81]. Similarly, high-mannose, complex type asialylation and the bi-, tri- and tetra-antennary sialylated structures are differentially expressed in early and late-stages of OvCa. Moreover, a combination of sialic acid ratio and CA 125 stratified early and late-stage OvCa patients with improved sensitivity and specificity of 89.6% and 100%, respectively, when compared to a CA125 sensitivity of 84.4% and 97% specificity when used as a single marker, as shown in Table 5 [78]. To affirm the place of glycan alterations in the possible staging of OvCa, a study of an OvCa mouse model reported an increase in sialylation with increasing tumor size, implying that sialylation is associated with OvCa progression and, therefore, likely to be useful in staging [86]. These findings imply that apart from its demonstrated potential to make an early diagnosis of OvCa, glycan-based biomarkers could also find applications in monitoring disease progression and staging of OvCa, which could eliminate the need to carry out surgical procedures to obtain a biopsy for histological examination and staging.

## 5. Challenges of Glycan Biomarker Discovery

Although there exist many opportunities in the field of glycomics with regard to the discovery of biomarkers including those of OvCa, it has not been without challenges that have delayed reaping the full benefits it offers. The glycome complexity is enormous and had previously made it extremely complicated to undertake glycan biomaker studies. However, the introduction of the new broad-spectrum high-throughput analytical technologies has sufficiently addressed the questions of identification and characterization of the glycans. Consequently, there is increased output of research activities in OvCa glycan-based biomarker discovery.

The very nature of OvCa heterogeneity is partly responsible for the delay in delivery of an efficient screening and diagnostic strategy. Ovarian tumors manifest in different phenotypes, molecular biology, etiology and tumor progression, each of them taking different disease paths and with varied outcomes [101]. However, recent modernization of high-throughput technologies has equipped glycomics research with high-precision MS analyzers that are helping to unravel the heterogeneity dilemma. Consequently, high possibilities of developing sets of glycan-based parameters that will aid in the screening and early diagnosis of OvCa irrespective of their originality are now in sight. Variations in sample handling (sample collection, shipment, storage and processing) are unlikely to be a major source of result variability as others we have previously shown [102,103].

Several glycan-biomarker studies on OvCa have suffered from the fate of low statistical power due to the often-low sample size used, leading to frequent less conclusive findings. The problem of the small sample size is partly attributed to the low global prevalence rates of OvCa, which may require a prolonged duration of research, especially in follow-up studies. The problem of low prevalence means that a successful screening strategy must meet an incredibly higher performance threshold to eliminate chances of unnecessary surgeries due to false-positive results. The dangers of surgeries occasioned by false-positive results may far outweigh the benefits that the patients derive from the expected early diagnosis of OvCa as some surgeries may become complicated and even fatal. In our view, the problem of sampling can be overcome by embracing the strategy of broad multi-site collaborative studies that will attain high numbers of study participants quickly and cut recruitment duration.

## 6. Conclusions

To conclude, the parallel development of instrumentation and high-throughput platforms has allowed the discovery of new glycome-based biomarkers in recent years. The ultimate aim being to find effective screening and early diagnostic strategies that will bring about a reduction in mortalities associated with late diagnosis of OvCa. To date, much of the research findings on glycomic-based OvCa biomarkers still fall within the discovery phase. As methods are affordable and non-invasive, glycan-based biomarkers have the potential to be clinically approved in the future but they are yet to be subjected through the rigor of validation in multi-center studies in order to pave way for clinical approval.

## Figures and Tables

**Table 2:** Serum total *N*-glycan biomarkers for ovarian cancer. ↑- upregulation, ↓- downregulation.

Glycosylation Trait	Regulation in OvCa	Reference
high-mannosylation	↓	[74,78,81,82,83]
hybrid structures	↓	[82]
branching		
- mono-, biantennary	↓	[78,82]
- triantennary	↑	[74,76,78,81,82,84]
- tetraantennary	↑	[76,78,81,82]
bisection	↓	[76,83]
	↑	[85]
Sialylation		
- asialylated structures	↓	[78]
- sialylated structures	↑	[74,76,78,81,86]
- ratio α2-3/α2-6	↑	[73,81,84]

**Table 3:** IgG glycopeptide biomarkers of ovarian cancer. ↑- upregulation, ↓- downregulation.

Potential Biomarker	Regulation in OvCa	References
agalactosylation	↑	[52,73,75,76,94]
core fucosylation	↑	[52,73]
IgG1 sialylation	↓	[52]

**Table 4:** Potential acute phase proteins *N*-glycan biomarkers for ovarian cancer. Le^X^, Lewis^X^.

Acute Phase Glycoprotein	Glycosylation Modulations	Reference
haptoglobin	antennarity, Le^x^, sialyl Le^x^	[73,97]
α-1-antichymotrypsin	antennarity, Le^x^, sialyl Le^x^	[73]
α-1-antitrypsin	Le^x^	[73]
α-1-acid glycoprotein	core-fucosylated bi-antennary digalactosylated sialyl Le^x^	[73,97]

**Table 5:** Diagnostic performances reported for glycan-based biomarkers.

Reference	Biomarker	Sensitivity/Specificity (%/%)	Cohort Size
[74]	GLYCOV (*N*-glycan score)	97/98.4	96
[81]	GLYCOV (*N*-glycan score)	95/97	73
[78]	CA-125 + sialic acid ratio	89.6/100	110
[77]	*N*-glycan score	80–90/70–83	80
[83]	*N*-glycan score	70/86.5	299
[94]	IgG_1_ + IgA + IgM + CA125 glycopeptide score	96.2/92.3	168
[75]	IgG agalactosylation ratio + CA125	90/84.6	58

## Data Availability

Not applicable.
